# Royal Westminster Ophthalmic Hospital

**Published:** 1830-07-01

**Authors:** 


### XXXII.

### ROYAL WESTMINSTER OPHTHALMIC HOSPITAL.

THE cases of cataract which have occurred since our last report have been numerous, and many of them highly interesting. We will notice some of the most remarkable both for their character and circumstances.

### CASE OF FLUID CATARACT.

The first case is the most remarkable, being that of a perfectly fluid cataract of the left eye, contained in a thickened capsule, which Mr. Guthrie said was exceedingly rare, and that he had never seen one so well marked as in this instance. The patient, John Jones, was 56 years of age, a gardener by trade. The cataract had existed two years—the iris was perfectly tremulous, and the pupil moderately dilated under the influence of the belladonna. On bending the head forward, the opaque body was seen to advance through the pupil, bulging towards the under part in consequence of its gravity, and was in some degree pendulous also, evidently indicating its fluid contents.

Mr. G. observed, that if the operation for extraction were performed, he had no doubt but the capsule would come out entire, and the greater part of the vitreous humour followed. The operation was performed in the presence of Mr. Thomas and a number of students, on the 23d of March, in the following manner.

The eyelids being separated, and the eye fixed with the fore-finger and thumb of the left hand, a two-edged cutting needle was introduced about two lines and a half behind the cornea, and placed in front of the capsule, which was immediately cut into, when a small quantity of white turbid fluid escaped into the anterior chamber; by this incision the capsule was evidently separated from its attachments, and beginning to descend;—no further attempts were made therefore to detach it, lest it should float in the vitreous humour. The needle was withdrawn, and the belladonna applied in order to keep the pupil as much dilated as possible.

10, p. m. He complained of pain in the head, but not in the eyes, and has vomited. Eight ounces of blood have been taken from the temple by cupping, which has relieved him. Pulv. jalapæ c. ʒj. st. sumendus.

24th. Pain returned about 4 o'clock this morning. Twelve ounces of blood were taken from the arm, which was repeated at 11 o'clock, and again to-night, producing syncope.

25th. He has passed a good night, and had no return of pain.

26th. Apparently well—wears a shade.

30th. Discharged. Cataract is entirely gone, and he is able to see nearly as well as with the other eye. The only appearance of disease remaining is the tremulous state of the iris.

### Case of Soft Cataract.

James Herring, æt. 25, applied to have an operation performed on his right eye, as the white spot in it prevented his getting a place in the police. The cataract was large, soft, and white, pressing against the pupil, which was a little dilated, and, in consequence of the pressure, shewing the black edge of the uvea. The iris had slightly altered in colour, and the patient could distinguish but very little light.

Mr. G. observed this was one of those cases in which the inflammation following the operation would, without doubt, be considerable, and require the most active treatment; and although the eye would recover its natural appearance, he hardly expected the man would obtain good sight.

Feb. 25th. A two-edged cutting needle was introduced behind the iris and carried forwards between it and the lens, with its flat side lying upon the capsule. The superior cutting edge being now turned towards the capsule, both it and the lens were completely divided, and this was frequently repeated until the lens was evidently divided into several small pieces. The needle was then withdrawn, and the belladonna applied.

5, p. m. Complained of pain in the eye and side of the head, upon which 24 ounces of blood were immediately taken from the arm, and afforded complete relief.

℞. Pulv. jalapæ, c. ʒj. st.

℞. Cal. gr. v.—Ext. colocynth, gr. v. post horas duas.

10, p. m. He has been cupped to 12 ounces, pain having returned.

26th. A return of pain took place at 2 o'clock, shooting across the forehead, when six ounces of blood were taken from the jugular vein, on which the patient fainted. He slept for several hours after this, and was quite free from pain in the afternoon. Bowels thoroughly open—belladonna plaister has been applied over the eye and forehead.

P.M. Pain having returned in the eye, he has been again bled to 10 ounces, and is quite relieved.

27th. Slept tolerably well during the night, and is free from pain.

Vespere. He has again complained of pain in his head and back, and has been ordered ʒj. of the vinum colchici.

March 1st. The pupil is well dilated—very little redness of the eye and no pain.

7th. He has complained to-day of a slight return of pain in the eye—cupped to ℥viij. The broken portions of the cataract evidently diminishing in size.

23d. The absorption of the cataract going on but slowly Mr. Guthrie thought it right to repeat the operation, but it was now evident that a great change had taken place in the portions of the lens, their consistence being now much greater. In this operation the whole of the portions were brought into the anterior chamber. The belladonna was repeated as before. 6 p. m. He has complained of pain in the head and eye for which he was bled to ℥xij. with immediate relief.

Eleven o'clock. Pain has returned and the abstraction of ℥viij. of blood has afforded him immediate relief.

24th. Passed a good night and free from pain.

Nine p. m. A return of pain relieved by the abstraction of ℥xiv. of blood.

25th. Quite free from pain, and going on very well. The portions of the lens are removing rapidly.

28th. A slight return of pain has taken place. C. c. temp. ad ℥viij.

From this time to the 20th of April no treatment was required beyond the belladonna. When he was discharged the pupil was perfectly clear and round; he saw much better than had been expected and immediately obtained his place as a police constable.

### Cases of Congenital Cataract.

Elizabeth Buchanan, æt. six months, admitted having cataract in both eyes from birth. The pupils being dilated with belladonna, and the eye fixed with a speculum, a needle was introduced through the sclerotic coat and the capsule divided which contained a small portion of soft lens—both were broken up as much as possible and pushed into the anterior chamber. The other eye was treated in the same manner. No inflammation ensued; great care was however taken to keep the pupils thoroughly dilated by the belladonna. Six weeks after this operation, that is in the beginning of November, the needle was again introduced in both eyes for the removal of a small portion of capsule. The belladonna was reapplied as before. No inflammation ensued, and the child was discharged cured—without a vestige of disease and apparently seeing very well.

Frederick Saene, æt. four, admitted November 10th, 1829. Cataract in both eyes. This child is also dumb and nearly deaf. November 11th. Mr. Guthrie broke up the cataracts of both eyes in a similar manner with the last case.

Nov. 21. The child has not suffered from the slightest degree of inflammation. The pupils are quite clear, and the child can see to run about, avoiding every thing in its way.

### Anomalous Cataract.

Ann Brunun, æt. 28, admitted having an anomalous cataract in the left eye. The opacity occupies only the inner half of the lens and capsule when the pupil is dilated and presents a very defined margin somewhat of a circular form. There are a few opaque spots on the other parts of the capsule, but the difference between the inner and outer half of it is very remarkable. She says that she suffered an attack of inflammation in this eye when she was two years old, and which she believes has caused this complaint, and wishes to have it removed on account of the deformity it occasions.

Dec. 2d. Mr. G. passed a two edged needle through the sclerotic coat, and into the capsule, on the division of which a substance something resembling white powder started forth, rendering the aqueous tumour very turbid, not however sufficiently so to prevent a complete division of the capsule, which cut like a piece of parchment. Belladonna ordered to be applied night and morning.

Dec. 13. She has suffered little pain since the operation, and it has not been necessary to abstract blood. One small piece of capsule alone remains in sight, which she does not consider any detriment, and says she can see a little with it, which she could not do before.

### Capsular Cataract after Injury.

Eliza Uyan, æt. six. Has a capsular cataract in the right eye, apparently from an injury—there being a small cicatrix at the upper margin of the cornea as if something had penetrated that membrane. The capsule was broken up on the 2d of March, and on the 13th she was discharged, the pupil being quite clear, and the sight tolerably good—no inflammation supervened, and the treatment was confined to purgatives and the application of the belladonna.

### Case of Hard Cataract.

Edward Thompson, æt. 40, was admitted April 24th, having lost the left eye, which was sunk in the head after an operation performed in the country two years ago—the right eye appeared perfectly sound, with the exception of a hard cataract.

Mr. Guthrie standing behind the patient fixed the eye-lids and the eye-ball with the fore and second finger of the left hand. He then carried the extracting knife across the cornea, completing the punctuation but not allowing it to cut its way completely out. The patient having slightly recovered from this, the guarded knife was passed through the opening, and its cutting edge being pushed forward divided the remaining portion of the cornea upwards. The capsule being now torn with the hook, the lens gradually advanced and was removed. The cut edges of the cornea were duly adjusted and the patient put to bed.

April 27th. There appears a slight threatening of inflammation, but the patient experiences no pain. C.c. ad ℥viij. temp.

From this time to May 16th he has not had an unfavourable symptom, and he is now ready to be discharged—the pupil being perfectly clear and his sight restored.

